# An updated “norepinephrine equivalent” score in intensive care as a marker of shock severity

**DOI:** 10.1186/s13054-023-04322-y

**Published:** 2023-01-20

**Authors:** Yuki Kotani, Annamaria Di Gioia, Giovanni Landoni, Alessandro Belletti, Ashish K. Khanna

**Affiliations:** 1 Department of Anesthesia and Intensive Care, IRCCS San Raffaele Scientific Institute, Via Olgettina 60, 20132 Milan, Italy; 2 School of Medicine, Vita-Salute San Raffaele University, Via Olgettina 58, 20132 Milan, Italy; 3 Department of Intensive Care Medicine, Kameda Medical Center, 929 Higashi-cho, Kamogawa, Chiba 296-8602 Japan; 4 Section on Critical Care Medicine, Department of Anesthesiology, Wake Forest Center for Biomedical Informatics, Perioperative Outcomes and Informatics Collaborative, Wake Forest University School of Medicine, Medical Center Boulevard, Winston-Salem, NC 27157 USA; 5 Outcomes Research Consortium, Cleveland, OH 44195 USA

**Keywords:** Norepinephrine, Norepinephrine equivalence, Vasopressor, Hemodynamic management, Vasopressin, Angiotensin II, Methylene blue

## Abstract

Vasopressors and fluids are the cornerstones for the treatment of shock. The current international guidelines on shock recommend norepinephrine as the first-line vasopressor and vasopressin as the second-line vasopressor. In clinical practice, due to drug availability, local practice variations, special settings, and ongoing research, several alternative vasoconstrictors and adjuncts are used in the absence of precise equivalent doses. Norepinephrine equivalence (NEE) is frequently used in clinical trials to overcome this heterogeneity and describe vasopressor support in a standardized manner. NEE quantifies the total amount of vasopressors, considering the potency of each such agent, which typically includes catecholamines, derivatives, and vasopressin. Intensive care studies use NEE as an eligibility criterion and also an outcome measure. On the other hand, NEE has several pitfalls which clinicians should know, important the lack of conversion of novel vasopressors such as angiotensin II and also adjuncts such as methylene blue, including a lack of high-quality data to support the equation and validate its predictive performance in all types of critical care practice. This review describes the history of NEE and suggests an updated formula incorporating novel vasopressors and adjuncts.

## Introduction

Shock is common and associated with mortality in patients admitted to intensive care units (ICUs) [1, 2]. In the physiological state, blood pressure is maintained within normal range by the interplay of three major mechanisms: the sympathetic nervous system, vasopressin system, and renin–angiotensin–aldosterone system [3]. However, in patients with vasodilatory shock, these homeostatic mechanisms are disturbed [4, 5]. When hypotension is not resolved solely by fluid resuscitation, vasopressor agents are the cornerstone of shock management to maintain adequate mean arterial pressure (MAP) [6, 7].

It is common among clinicians and researchers to use the dose of vasopressor agents to grade the severity of shock. Norepinephrine has been recommended as the first-line vasopressor since 2004 [8], and the latest guidelines suggest starting vasopressin on top of norepinephrine when a target MAP is not achieved [9]. On the other hand, many uncertainties remain in the care of shock, including how and when to start other vasopressor agents [10]. Although new vasopressors (e.g., angiotensin II, methylene blue) have become popular in intensive care practice, there is little evidence from high-quality randomized trials and no clear recommendation in the guidelines to guide clinical decisions on when and how to initiate these new vasopressor agents so far [11]. In addition, there are conditions where these and other vasopressors are used with or without norepinephrine, such as catecholamine-resistant vasodilatory shock [12]. Since different vasopressors have different pharmacological characteristics and effects on hemodynamics, a calculation formula that reflects the potency of each vasopressor is frequently necessary to describe the degree of vasopressor support in a standardized manner. This is especially true when designing and conducting clinical trials.

Norepinephrine equivalence (NEE) is a scale to quantify vasopressor exposure, which converts the dose of each vasopressor equivalent to that of norepinephrine. NEE has been used in clinical trials to set an inclusion criterion, define trial protocols, report baseline characteristics, and assess outcomes [13–19]. For example, inclusion criteria in the Angiotensin II for the treatment of high-output shock 3 (ATHOS-3) trial necessitated a norepinephrine equivalence calculator for patient enrollment at doses > 0.2 µg/kg/min of NEE. However, the major issue with NEE is that there is no standardized method for measuring the potency of vasopressors. As a result, there are several different calculation formulas for NEE [12, 13, 15, 16, 20–23]. Inconsistent calculation methods for NEE will make it difficult to compare or interpret the results between clinical studies. In addition, whenever a new vasopressor enters intensive care practice, there is a need to update the last NEE formula.

This review aims to describe the evolution of NEE, its utility in clinical research and practice, its pitfalls, and future perspectives and opportunities with a proposal to produce an updated version of the score.

## Evolution of calculation formulas

In 1995, the history of quantifying the amount of hemodynamic support began when the inotrope score (IS) was developed for neonates after congenital cardiac surgery [24]. The IS was calculated as dopamine dose (µg/kg/min) + dobutamine dose (µg/kg/min) + 100 × epinephrine dose (µg/kg/min) [25]. In 2002, the first attempt to integrate the dose of different vasopressors, including norepinephrine, into one scale, especially in adult septic shock, defined NEE as norepinephrine dose (µg/min) + epinephrine dose (µg/min) + 1/4 × dopamine dose (µg/kg/min) [22]. In 2008, the VASST (Vasopressin and septic shock trial) study modified the previous NEE dose as norepinephrine dose (µg/min) + 1/2 × dopamine dose (µg/kg/min) + epinephrine dose (µg/min) + 1/10 × phenylephrine dose (µg/min) [13]. Reflecting the increasing use of vasopressin after the VASST study in septic shock, different NEE formulas, which incorporate vasopressin, started to be used [13–15, 17–19], which are slightly different from each other. A clinical trial assessing the effect of selepressin, a cyclic nonapeptide vasopressin analog, used a unique NEE equation without vasopressin due to the trial design with strict restriction of vasopressin use [16]. However, selepressin is not commercially available since this trial failed to show any clinically relevant superiority in patients assigned to selepressin over a placebo. Since the conversion ratio of each calculation is based on unclear evidence, a recent scoping review proposed another approach to determine a calculation formula [20]. The authors extracted the conversion ratios from 21 clinical trials comparing the equipotency of different vasopressors to achieve the target blood pressure. With the data of the eligible 21 studies, the scoping review suggested the following formula: norepinephrine dose (µg/kg/min) + epinephrine dose (µg/kg/min) + 1/10 × phenylephrine dose (µg/kg/min) + 1/100 × dopamine dose (µg/kg/min) + 1/8 × metaraminol dose (µg/kg/min) + 2.5 × vasopressin dose (U/min) + 10 × angiotensin II dose (µg/kg/min).

Table 1 summarizes different NEE equations reported in the literature so far.

**Table 1:** Summary of norepinephrine equivalent formulas

Author	Year	Journal	Calculation
Patel et al. [22]	2002	Anesthesiology	Norepinephrine dose (µg/min) + epinephrine dose (µg/min) + 1/4 × dopamine dose (µg/kg/min)
Russell et al. [13] (VASST)	2008	New England Journal of Medicine	Norepinephrine dose (µg/min) + 1/2 × dopamine dose (µg/kg/min) + epinephrine dose (µg/min) + 1/10 × phenylephrine dose (µg/min)
Brown et al. [12]	2013	Chest	Norepinephrine dose (µg/kg/min) + epinephrine dose (µg/kg/min) + 1/100 × dopamine dose (µg/kg/min) + 5 × vasopressin dose (U/min) + 0.45 × phenylephrine dose (µg/kg/min)
Ralib et al. [23]	2013	Clinical Nephrology	Norepinephrine dose (μg/min) + 500 × vasopressin dose (U/min) + epinephrine dose (μg/min) + 1/3 × phenylephrine dose (μg/min) + 1/100 × dopamine dose (μg/min)
Gutsche et al. [21]	2017	Anesthesia & Analgesia	Norepinephrine dose (μg/min) + 1/2 × dopamine dose (μg/kg/min) + epinephrine dose (μg/min) + 1/10 × phenylephrine dose (μg/min) + 200 × vasopressin dose (U/min)
Khanna et al. [15] (ATHOS-3)	2017	New England Journal of Medicine	Norepinephrine dose (µg/kg/min) + epinephrine dose (µg/kg/min) + 1/150 × dopamine dose (µg/kg/min) + 1/10 × phenylephrine dose (µg/kg/min) + 2.5 × vasopressin dose (U/min)
Laterre et al. [16] (SEPSIS-ACT)	2019	JAMA	Norepinephrine dose (µg/min) + epinephrine dose (µg/min) + 1/100 × dopamine dose (µg/min) + 1/2.2 × phenylephrine dose (µg/kg/min)
Goradia et al. [20]	2021	Journal of Critical Care	Norepinephrine dose (µg/kg/min) + epinephrine dose (µg/kg/min) + 1/10 × phenylephrine dose (µg/kg/min) + 1/100 × dopamine dose (µg/kg/min) + 1/8 × metaraminol (µg/kg/min) + 2.5 × vasopressin dose (U/min) + 10 × angiotensin II dose (µg/kg/min)
Our manuscript	2022		Norepinephrine dose (µg/kg/min) + epinephrine dose (µg/kg/min) + 1/100 × dopamine dose (µg/kg/min) + 0.06 × phenylephrine dose (µg/kg/min) + 2.5 × vasopressin dose (U/min) + 0.0025 × angiotensin II dose (ng/kg/min) + 10 × terlipressin dose (µg/kg/min) + 0.2 × methylene blue dose (mg/kg/h) + 1/8 × metaraminol dose (µg/kg/min) + 0.02 × hydroxocobalamin dose (g) + 0.4 × midodrine dose (µg/kg/min)

## Need for using norepinephrine equivalence

NEE allows us to combine the dose of different vasopressor agents into a single scale, and this characteristic is advantageous when patients receive multiple vasopressors simultaneously. Although norepinephrine is the first-line vasopressor in critical care, adding secondary agents is suggested when norepinephrine alone cannot attain the target pressure or when the norepinephrine dose required to achieve the target MAP becomes excessive [9]. From a pathophysiological point of view, several different mechanisms are implicated in vasodilatory hypotension, such as inadequate secretion of vasopressin from the posterior pituitary and down-regulation of angiotensin receptors, which would make the use of non-catecholamine vasopressor along with norepinephrine reasonable [3]. NEE would also help to quantitatively compare the severity of shock when norepinephrine is not readily available, e.g., in norepinephrine shortage [26] or low-middle income countries [27].

**Fig. 1:**
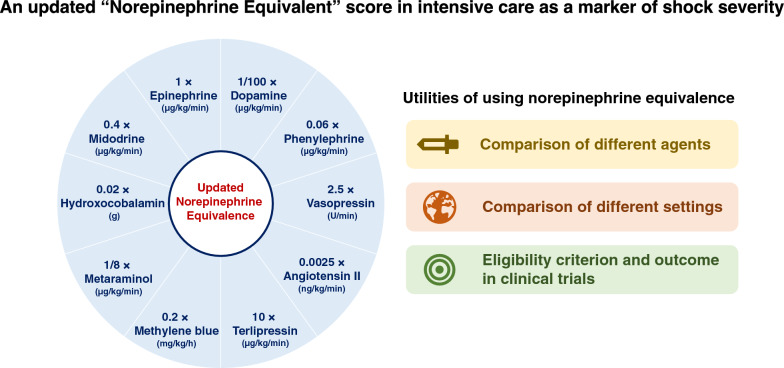
Visual summary of an updated norepinephrine equivalent score and need for using norepinephrine equivalence

Furthermore, NEE can serve as an eligibility criterion in clinical trials. Although clinical research, especially randomized controlled trials, should determine which patient is eligible for enrolment accurately and objectively, the precise definition of eligibility criteria is not always easy. For example, it would be difficult to decide whether a patient on norepinephrine of 0.3 µg/kg/min and vasopressin 0.03 U/min is eligible when “receiving norepinephrine ≥ 0.4 µg/kg/min” is listed in the inclusion criteria of a randomized trial. NEE can overcome this challenge by standardizing the potency of vasopressors, and several randomized trials used NE as an inclusion criterion [13, 15]. NEE can also be the primary endpoint, especially in feasibility, pilot trials, and observational studies. Figure 1 summarizes the need for using NEE score.

A novel measure of hypotension using NEE has been recently proposed, i.e., the ratio of MAP and NEE [28]. Like PaO_2_/FiO_2_ ratio as a measure of oxygenation, MAP/NEE can be used as a measure of vasopressor responsiveness and severity of shock.

## Pitfalls

NEE has several pitfalls. First, with scarce evidence, the conversion ratio for each vasopressor agent is determined arbitrarily, either comparing the dose needed to achieve a target MAP or estimating the reduction in norepinephrine dose when used in combination. This drawback is especially important given the recent evidence supporting a multimodal vasopressor approach [3]. The calculation ratio in NEE (e.g., 1/100–1/150 for dopamine in some established formulas) is generally defined according to the equipotency of each vasopressor compared to norepinephrine to achieve the same MAP target. As a result, due to the different hemodynamic effects of vasopressors (e.g., vasopressors with inotropic effect or pure vasoconstrictors) and the complex interaction between vascular tone, volume status, and cardiac contractility, similar MAPs may correspond to very different hemodynamic profile despite comparable NEE. On the other hand, NEE allows clinicians to compare the hemodynamic, microcirculatory, or metabolic effects of different vasopressor agents by adjusting the dose of each drug in terms of vasoconstrictive effects.

Second, NEE may sometimes not reflect the total amount of hemodynamic support. Since NEE only considers vasopressor effects, NEE fails to measure the effect of other hemodynamic interventions, such as mechanical circulatory support and drugs with predominantly inotropic profiles. For example, consider a patient with severe low cardiac output syndrome who receives veno-arterial extracorporeal membrane support and moderate to a high dose of inotropes in addition to low-dose norepinephrine. In that case, it will be obvious that the NEE for this patient is disproportionately low compared to the total hemodynamic support. However, it is quite challenging to integrate the intensity of mechanical circulatory support or inotropic agents with vasopressors. In general, MAP is the single measure of efficacy to guide vasopressors, while mechanical circulatory support or inotropes require other parameters (e.g., cardiac index—CI) on top of MAP, where it becomes difficult to ascertain how much a combination of vasopressor and inotrope contributes to MAP or CI. Therefore, NEE should be used and interpreted cautiously in studies, including patients requiring mechanical circulatory support or high-dose inotropes. Although a scoping review proposed a calculation formula based on the available evidence on the equipotency of different vasopressors, the small number of included studies on each vasopressor limits its generalizability [20].

Third, we need to renew the NEE equation whenever a new vasopressor emerges. Any new vasopressor comes with less evidence that, in most cases, is not enough to allow an accurate construct of a validated and updated NEE equation. This questions our traditional approach with complication derivations of the NEE equation and pushes us to think to simplify this process.

## Proposed updated norepinephrine equivalent score

We propose an updated NEE equation based on the best available evidence on the equipotency of various vasopressors. Two randomized controlled trials comparing epinephrine and norepinephrine found that the dose necessary to achieve the same MAP target was similar between the two vasopressors [29, 30]. Therefore, we assigned 1 as a conversion ratio to epinephrine.

Most previous NEE formulas used 1/100 or 1/150 as the conversion ratio for dopamine [12, 15, 16, 20, 23]. Two randomized trials showed that approximately 80 and 140 times the dose of dopamine was required to reach the same MAP target when compared with norepinephrine, respectively [31, 32]. These results were followed by the largest randomized trial comparing dopamine and norepinephrine (SOAP II trial), which demonstrated the potency of dopamine is 1/100 times that of norepinephrine [33]. Accordingly, we assigned 1/100 to the conversion ratio for dopamine.

A small, non-randomized study in septic shock patients found that 3.2 µg/kg/min of phenylephrine was equivalent to 0.2 µg/kg/min to obtain MAP ≥ 65 mmHg [34]. A randomized trial in patients under spinal anesthesia found 39.1 µg/min of phenylephrine was equivalent to 2.4 µg/min of norepinephrine [35]. Based on these studies, 0.06 was assigned to the conversion ratio of phenylephrine.

There are two large randomized controlled trials comparing vasopressin and norepinephrine in septic shock [13, 36]. In one trial, 0.03 U/min of vasopressin corresponded to 7.5 µg/min of norepinephrine [13], while another trial found that 0.06 U/min of vasopressin resulted in norepinephrine infusion rate by 0.15 µg/kg/min [36]. Thus, the conversion ratio of 2.5 would be reasonable for vasopressin.

Since ATHOS-3 is the only multicenter randomized controlled trial to assess the equipotency of angiotensin II in intensive care settings, we adopted the result of this trial to calculate a conversion factor for angiotensin II. This trial reported that 20 ng/kg/min of angiotensin II infusion resulted in a mean decrease of 0.05 µg/kg/min of norepinephrine compared with the placebo to maintain a target MAP of 75 mmHg or 10 mmHg greater than baseline in the first 3 h of drug initiation, and a target MAP of 65 mmHg afterward. Thus, we applied a correction factor of 0.0025 to the angiotensin II dose in ng/kg/min.

Similarly, data from a recent multicenter randomized trial comparing terlipressin with norepinephrine in septic shock [37], we applied a correction factor of 10 to the terlipressin dose in μg/kg/min.

A randomized trial compared metaraminol and norepinephrine in septic shock [38]. Based on the findings of this trial suggesting 2.5 μg/kg/min of metaraminol corresponded to 0.3 μg/kg/min of norepinephrine, we defined a correction factor of 1/8 to metaraminol dose in μg/kg/min.

A recent randomized trial found that 5 g of hydroxocobalamin reduced norepinephrine by 0.08 µg/kg/min [39], which led us to apply 0.02 as a correction factor to hydroxocobalamin dose in g.

A randomized trial comparing oral midodrine with intravenous norepinephrine found that 30 mg/day of midodrine reduced 73 mg of norepinephrine during six days in septic shock [40], which gave a correction factor of 0.4 to midodrine dose in μg/kg/min.

On the other hand, there is no randomized trial comparing the potency of methylene blue with that of other vasoconstrictors. A single-center randomized trial assessing the efficacy of methylene blue in septic shock [41] reported that the doses for methylene blue infusion ranged from 0.25 to 2 mg/kg/h. Accordingly, we arbitrarily applied a correction factor of 0.2 to the dose of methylene blue in mg/kg/h.

Therefore, we have updated the NEE equation incorporating vasoconstrictors commonly used in recent years. Figure 1 and Table 1 describe our final modified NEE calculation formula. If we compare our formula with the one by Goradia et al. [20], the correction factors for angiotensin II are inconsistent even after the adjustment of the unit used (2.5 vs. 10). The main reason for the difference is the data source for the correction factor estimation. While Goradia et al. used a pilot single-center randomized trial published in 2012 in intensive care unit settings [42], we used a subsequent multicenter randomized trial published in 2017 [15]. Since the pilot trial was small in sample size and had baseline imbalances between angiotensin and placebo arms, we used only the larger trial to better estimate the equipotency of angiotensin II.

All the studies we used to update the NEE formula are listed in Table 2.

**Table 2:** Studies to determine the conversion ratio of each vasopressor agent in our updated NEE formula

Author	Year	Journal	Equipotency with norepinephrine
*Epinephrine*			
Myburgh et al. [29]	2008	Intensive Care Med	There was no difference in the maximal daily infusion dose between norepinephrine and epinephrine
Annane et al. [30]	2007	Lancet	The doses of vasopressors needed to achieve the mean arterial pressure target were not different between epinephrine and norepinephrine
*Dopamine*			
Marik et al. [32]	1994	JAMA	26 µg/kg/min of dopamine was equivalent to 0.18 µg/kg/min of norepinephrine
De Backer et al. [31]	2003	Crit Care Med	The median dose of dopamine was 15 µg/kg/min, while that of norepinephrine was 0.18 µg/kg/min
De Backer et al. [33]	2010	New England Journal of Medicine	The dose of dopamine was consistently 100 times as much as that of norepinephrine during seven days after randomization
*Phenylephrine*			
Reinelt et al. [34]	1999	Crit Care Med	3.2 µg/kg/min of phenylephrine was equipotent to 0.2 µg/kg/min of norepinephrine
Ngan Kee et al. [35]	2015	Anesthesiology	39.1 µg/min of phenylephrine was equivalent to 2.4 µg/min of norepinephrine
*Vasopressin*			
Russell et al. [13] (VASST)	2008	New England Journal of Medicine	The addition of 0.03 U/min of vasopressin reduced 7.5 µg/min of norepinephrine
Gordon et al. [36]	2016	JAMA	The addition of 0.06 U/min of vasopressin reduced norepinephrine dose by 0.15 µg/kg/min
*Other vasopressor agents*			
Khanna et al. [15]	2017	New England Journal of Medicine	20 ng/kg/min of angiotensin II infusion resulted in a mean reduction of 0.05 µg/kg/min of norepinephrine compared with the placebo
Liu et al. [37]	2018	Intensive Care Med	The dose of terlipressin required to reach the mean arterial pressure target was ten times lower than that norepinephrine dose
Natalini et al. [38]	2005	Intensive Care Med	2.5 μg/kg/min of metaraminol corresponded to 0.3 μg/kg/min of norepinephrine
Patel et al. [39]	2022	Chest	5 g of hydroxocobalamin reduced norepinephrine requirement by 0.08 µg/kg/min
Adly et al. [40]	2022	Ir J Med Sci	30 mg/day of midodrine reduced 73 mg of norepinephrine during six days
Kirov et al. [41]	2001	Crit Care Med	Methylene blue infusion ranged from 0.25 to 2 mg/kg/h, while norepinephrine ranged from 0.1 to 0.7 µg/kg/min

## Future perspectives

There are several unanswered questions concerning NEE. The current trend toward a multimodal vasopressor strategy will require a valuable measure to standardize the total amount of vasoconstrictor therapy. Although NEE is a helpful measure of vasopressor support, regular updates, e.g., the use of novel vasoconstrictors, are necessary. Since most correction ratios are arbitrarily determined, validation studies are also required to evaluate the predictive performance of NEE for worse clinical outcomes.

## Conclusions

Since its emergence in 2002, NEE has been increasingly used in intensive care research. Its importance will further increase if the catecholamine-sparing vasopressor strategy becomes prevalent. Regular renewal and validation are necessary to update NEE in line with clinical practice.

## Data Availability

Further information is available from the corresponding authors upon reasonable request.

## Abbreviations

ATHOS-3: Angiotensin II for the treatment of high-output shock 3
CI: Cardiac index
ICU: Intensive care unit
MAP: Mean arterial pressure
NEE: Norepinephrine equivalence
VASST: Vasopressin and septic shock trial
