# Correction: An updated “norepinephrine equivalent” score in intensive care as a marker of shock severity

**DOI:** 10.1186/s13054-025-05250-9

**Published:** 2025-03-07

**Authors:** Yuki Kotani, Annamaria Di Gioia, Giovanni Landoni, Alessandro Belletti, Ashish K. Khanna

**Affiliations:** 1 Department of Anesthesia and Intensive Care, IRCCS San Raffaele Scientific Institute, Via Olgettina 60, 20132 Milan, Italy; 2 School of Medicine, Vita-Salute San Raffaele University, Via Olgettina 58, 20132 Milan, Italy; 3 Department of Intensive Care Medicine, Kameda Medical Center, 929 Higashi‑cho, Kamogawa, Chiba 296‑8602 Japan; 4 Section on Critical Care Medicine, Department of Anesthesiology, Wake Forest Center for Biomedical Informatics, Perioperative Outcomes and Informatics Collaborative, Wake Forest University School of Medicine, Medical Center Boulevard, Winston‑Salem, NC 27157 USA; 5 Outcomes Research Consortium, Cleveland, OH 44195 USA

**Correction: Critical Care (2023) 27:29** 10.1186/s13054-023-04322-y

Following publication of the original article [1], the authors would like to correct the correction rate for metaraminol which is 1/8 under the heading Proposed updated norepinephrine equivalent score, Figure 1 and Table 1.

The sentences currently reads:

A randomized trial compared metaraminol and norepinephrine in septic shock [38]. Based on the findings of this trial suggesting 2.5 μg/kg/min of metaraminol corresponded to 0.3 μg/kg/min of norepinephrine, we defined a correction factor of 8 to metaraminol dose in μg/kg/min.

The sentences should read:

A randomized trial compared metaraminol and norepinephrine in septic shock [38]. Based on the findings of this trial suggesting 2.5 μg/kg/min of metaraminol corresponded to 0.3 μg/kg/min of norepinephrine, we defined a correction factor of 1/8 to metaraminol dose in μg/kg/min.

The incorrect Figure 1:

**Fig. 1:**
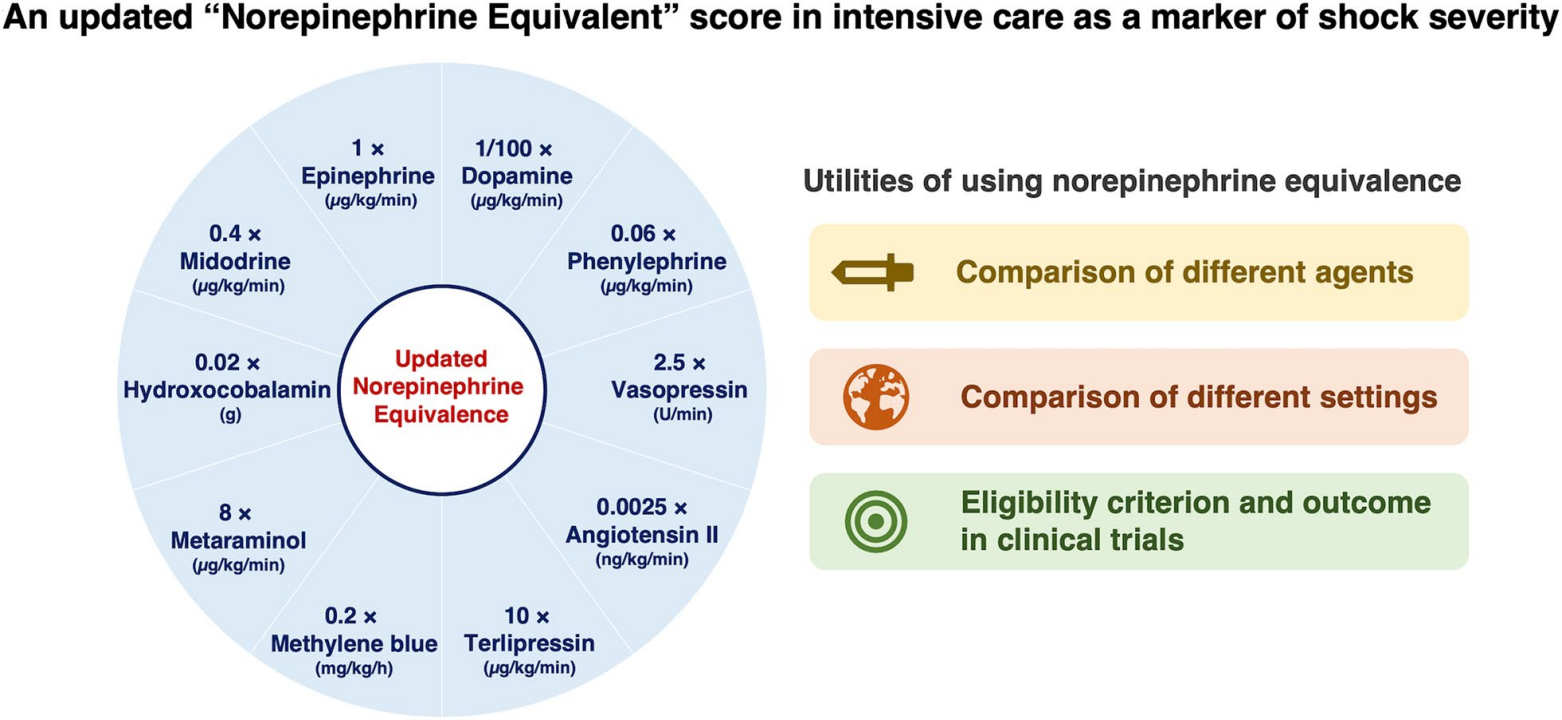
Visual summary of an updated norepinephrine equivalent score and need for using norepinephrine equivalence

The correct Figure 1:

**Fig. 1:**
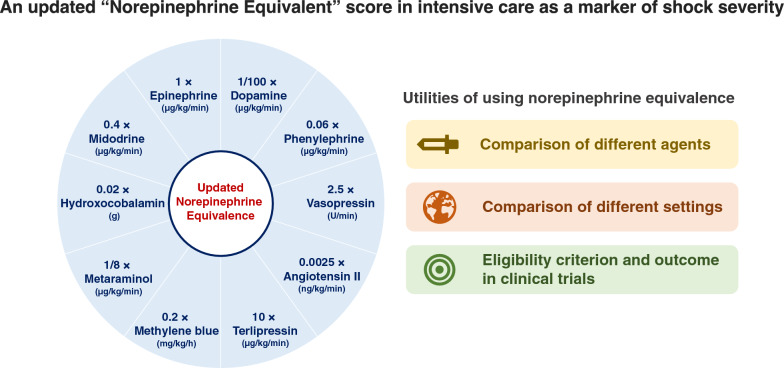
Visual summary of an updated norepinephrine equivalent score and need for using norepinephrine equivalence

The incorrect Table 1:

**Table 1:** Summary of norepinephrine equivalent formulas

Author	Year	Journal	Calculation
Patel et al. [22]	2002	Anesthesiology	Norepinephrine dose (µg/min) + epinephrine dose (µg/min) + 1/4 × dopamine dose (µg/kg/min)
Russell et al. [13] (VASST)	2008	New England Journal of Medicine	Norepinephrine dose (µg/min) + 1/2 × dopamine dose (µg/kg/min) + epinephrine dose (µg/min) + 1/10 × phenylephrine dose (µg/min)
Brown et al. [12]	2013	Chest	Norepinephrine dose (µg/kg/min) + epinephrine dose (µg/kg/min) + 1/100 × dopamine dose (µg/kg/min) + 5 × vasopressin dose (U/min) + 0.45 × phenylephrine dose (µg/kg/min)
Ralib et al. [23]	2013	Clinical Nephrology	Norepinephrine dose (μg/min) + 500 × vasopressin dose (U/min) + epinephrine dose (μg/min) + 1/3 × phenylephrine dose (μg/min) + 1/100 × dopamine dose (μg/min)
Gutsche et al. [21]	2017	Anesthesia & Analgesia	Norepinephrine dose (μg/min) + 1/2 × dopamine dose (μg/kg/min) + epinephrine dose (μg/min) + 1/10 × phenylephrine dose (μg/min) + 200 × vasopressin dose (U/min)
Khanna et al. [15] (ATHOS-3)	2017	New England Journal of Medicine	Norepinephrine dose (µg/kg/min) + epinephrine dose (µg/kg/min) + 1/150 × dopamine dose (µg/kg/min) + 1/10 × phenylephrine dose (µg/kg/min) + 2.5 × vasopressin dose (U/min)
Laterre et al. [16] (SEPSIS-ACT)	2019	JAMA	Norepinephrine dose (µg/min) + epinephrine dose (µg/min) + 1/100 × dopamine dose (µg/min) + 1/2.2 × phenylephrine dose (µg/kg/min)
Goradia et al. [20]	2021	Journal of Critical Care	Norepinephrine dose (µg/kg/min) + epinephrine dose (µg/kg/min) + 1/10 × phenylephrine dose (µg/kg/min) + 1/100 × dopamine dose (µg/kg/min) + 1/8 × metaraminol (µg/kg/min) + 2.5 × vasopressin dose (U/min) + 10 × angiotensin II dose (µg/kg/min)
Our manuscript	2022		Norepinephrine dose (µg/kg/min) + epinephrine dose (µg/kg/min) + 1/100 × dopamine dose (µg/kg/min) + 0.06 × phenylephrine dose (µg/kg/min) + 2.5 × vasopressin dose (U/min) + 0.0025 × angiotensin II dose (ng/kg/min) + 10 × terlipressin dose (µg/kg/min) + 0.2 × methylene blue dose (mg/kg/h) + 8 × metaraminol dose (µg/kg/min) + 0.02 × hydroxocobalamin dose (g) + 0.4 × midodrine dose (µg/kg/min)

The correct Table 1:

**Table 1:** Summary of norepinephrine equivalent formulas

Author	Year	Journal	Calculation
Patel et al. [22]	2002	Anesthesiology	Norepinephrine dose (µg/min) + epinephrine dose (µg/min) + 1/4 × dopamine dose (µg/kg/min)
Russell et al. [13] (VASST)	2008	New England Journal of Medicine	Norepinephrine dose (µg/min) + 1/2 × dopamine dose (µg/kg/min) + epinephrine dose (µg/min) + 1/10 × phenylephrine dose (µg/min)
Brown et al. [12]	2013	Chest	Norepinephrine dose (µg/kg/min) + epinephrine dose (µg/kg/min) + 1/100 × dopamine dose (µg/kg/min) + 5 × vasopressin dose (U/min) + 0.45 × phenylephrine dose (µg/kg/min)
Ralib et al. [23]	2013	Clinical Nephrology	Norepinephrine dose (μg/min) + 500 × vasopressin dose (U/min) + epinephrine dose (μg/min) + 1/3 × phenylephrine dose (μg/min) + 1/100 × dopamine dose (μg/min)
Gutsche et al. [21]	2017	Anesthesia & Analgesia	Norepinephrine dose (μg/min) + 1/2 × dopamine dose (μg/kg/min) + epinephrine dose (μg/min) + 1/10 × phenylephrine dose (μg/min) + 200 × vasopressin dose (U/min)
Khanna et al. [15] (ATHOS-3)	2017	New England Journal of Medicine	Norepinephrine dose (µg/kg/min) + epinephrine dose (µg/kg/min) + 1/150 × dopamine dose (µg/kg/min) + 1/10 × phenylephrine dose (µg/kg/min) + 2.5 × vasopressin dose (U/min)
Laterre et al. [16] (SEPSIS-ACT)	2019	JAMA	Norepinephrine dose (µg/min) + epinephrine dose (µg/min) + 1/100 × dopamine dose (µg/min) + 1/2.2 × phenylephrine dose (µg/kg/min)
Goradia et al. [20]	2021	Journal of Critical Care	Norepinephrine dose (µg/kg/min) + epinephrine dose (µg/kg/min) + 1/10 × phenylephrine dose (µg/kg/min) + 1/100 × dopamine dose (µg/kg/min) + 1/8 × metaraminol (µg/kg/min) + 2.5 × vasopressin dose (U/min) + 10 × angiotensin II dose (µg/kg/min)
Our manuscript	2022		Norepinephrine dose (µg/kg/min) + epinephrine dose (µg/kg/min) + 1/100 × dopamine dose (µg/kg/min) + 0.06 × phenylephrine dose (µg/kg/min) + 2.5 × vasopressin dose (U/min) + 0.0025 × angiotensin II dose (ng/kg/min) + 10 × terlipressin dose (µg/kg/min) + 0.2 × methylene blue dose (mg/kg/h) + 1/8 × metaraminol dose (µg/kg/min) + 0.02 × hydroxocobalamin dose (g) + 0.4 × midodrine dose (µg/kg/min)

The given sentences under the heading Proposed updated norepinephrine equivalent score, Figure 1 and Table 1 have been updated in this correction article and the original article [1] has been corrected.
